# The *Caenorhabditis elegans* INX‐4/Innexin is required for the fine‐tuning of temperature orientation in thermotaxis behavior

**DOI:** 10.1111/gtc.12745

**Published:** 2020-01-31

**Authors:** Satomi Tsukamoto, Taishi Emmei, Shunji Nakano, Nana Nishio, Hiroyuki Sasakura, Ikue Mori

**Affiliations:** ^1^ Division of Biological Science Graduate School of Science Nagoya University Nagoya Japan; ^2^ Neuroscience Institute Graduate School of Science Nagoya University Nagoya Japan; ^3^ Present address: Department of Physiology School of Medicine The University of Tokyo Tokyo Japan; ^4^ Present address: School of Medicine Aichi Medical University Nagakute Japan

**Keywords:** *C. elegans*, innexin, memory, thermotaxis

## Abstract

Innexins in invertebrates are considered to play roles similar to those of connexins and pannexins in vertebrates. However, it remains poorly understood how innexins function in biological phenomena including their function in the nervous systems. Here, we identified *inx‐4*, a member of the innexin family in *C. elegans*, by a forward screening of thermotaxis‐defective mutants. The *inx‐4* mutants exhibited abnormal migration to a temperature slightly higher than the cultivation temperature, called mild thermophilic behavior. Rescue experiments revealed that INX‐4 acts in the major thermosensory neuron AFD to regulate thermotaxis behavior. INX‐4::GFP fusion protein localized exclusively along axons in AFD neurons. In addition, over‐expression of INX‐4 in AFD neurons induced a cryophilic behavior, which is opposite to *inx-4* mutants. Our findings suggest that INX‐4/Innexin in AFD may fine‐tune the execution of thermotaxis behavior when moving to desired temperatures.

## Graphical abstract

Innexins in invertebrates are considered to play roles similar to those of connexins and pannexins in vertebrates. Here, we identified *inx‐4*, a member of the innexin family in *C. elegans*, by a forward screening of thermotaxis‐defective mutants. Our findings suggest that *inx‐4*/Innexin may fine‐tune the execution of thermotaxis behavior when moving to desired temperatures.

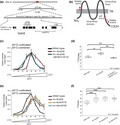

## 1 INTRODUCTION

Innexins in invertebrates are considered to have similar topology and functional properties to those of connexins and pannexins in vertebrates (Barbe, Monyer, & Bruzzone, 2006; Beyer, & Berthoud, 2018; Phelan & Starich, 2001; Simonsen, Moerman, & Naus, 2014). Connexins and innexins are known to form gap junctions by docking of hemichannels in adjacent cells and implicated in exchange of ions and small signaling molecules between cells (Phelan, 2005; Söhl, Maxeiner, & Willecke, 2005). The human genome contains 21 connexins and 3 pannexins (Penuela, Harland, Simek, & Laird, 2014; Söhl & Willecke, 2003), whereas the *Caenorhabditis elegans* and *Drosophila melanogaster* genomes have 25 and 8 innexins, respectively (Phelan & Starich, 2001; Stebbings et al., 2002). Although in vivo neuronal expressions of innexins are documented comprehensively in *C. elegans* (Altun, Chen, Wang, & Hall, 2009; Bhattacharya, Aghayeva, Berghoff, & Hobert, 2019), their roles in biological processes are still poorly understood.

*Caenorhabditis elegans* exhibits food‐associated thermotaxis behavior, where animals memorize information of environmental temperatures depending on the presence or absence of food (Mohri et al., 2005). When wild‐type animals cultivated at a certain temperature with food are placed on a food‐free plate subjected to a thermal gradient, these conditioned animals migrate to the previous cultivation temperature. On the other hand, animals that have been cultivated without food learn to disperse on the thermal gradient (Nishio et al., 2012). This food‐dependent behavioral paradigm called thermotaxis provides a great opportunity to study the functions of the nervous system, together with the genetic tools and the classical wiring diagram available in *C. elegans*.

Here, we found that INX‐4, a member of the innexin family in *C. elegans*, acts in the major thermosensory neuron, through which the animals execute proper thermotaxis behavior. By conducting a forward genetic screening of thermotaxis‐defective mutants, we isolated the *nj24* mutation exhibiting intriguing phenotypes. Well‐fed wild‐type animals migrate to the cultivation temperature on a thermal gradient. By contrast, well‐fed *nj24* mutants exhibited abnormal migration to a temperature slightly higher than the cultivation temperature. Furthermore, wild‐type animals that had been cultivated in food‐deprived condition dispersed on the gradient, whereas starved *nj24* mutants migrated toward a much higher temperature than the cultivation temperature. SNP mapping revealed that the *nj24* mutation is a mis‐sense mutation (Gly to Asp) in the putative seventh exon of the *inx‐4* gene encoding the innexin protein INX‐4. Rescue experiments revealed that INX‐4 acts only in the major thermosensory neuron to control thermotaxis. Animals over‐expressing INX‐4 in thermosensory neurons exhibited a cryophilic behavior, which is opposite to *inx‐4* mutant. These results indicate that INX‐4 in AFD has a unique and novel role to fine‐tune temperature orientation in thermotaxis.

## 2 RESULTS

### 2.1 *nj24* mutants showed a defective thermotaxis behavior

**Figure 1:**
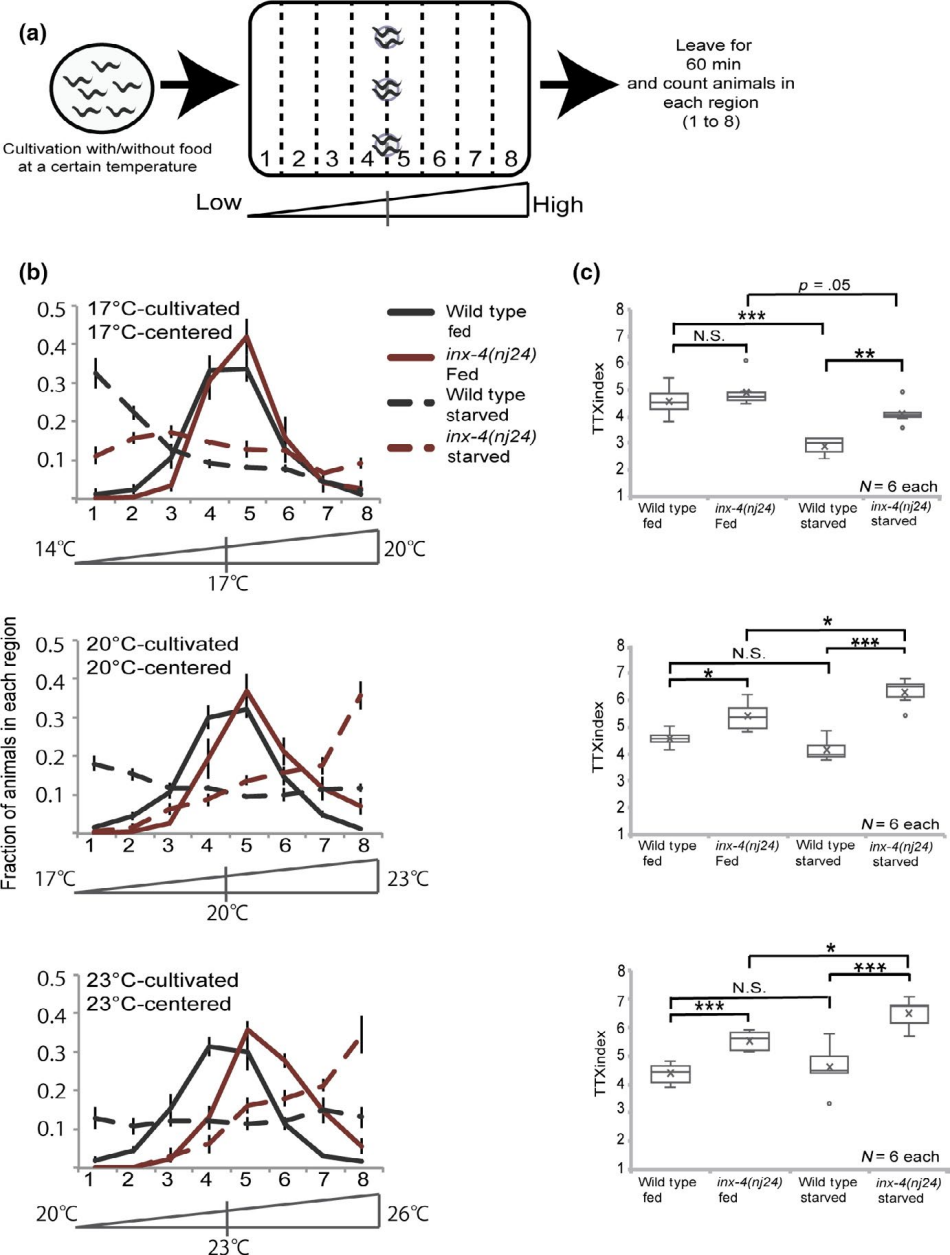
*nj24* mutants showed a defect in thermotaxis behavior in both fed and starved conditions. (a) Procedure for thermotaxis assay. Animals cultivated at a certain temperature were placed on the temperature gradient. After 60 min, the number of animals in each region (1–8) was counted. (b, c) Distribution of well‐fed and starved wild‐type animals and *nj24* mutants cultivated at 17°C (upper), 20°C (middle) or 23°C (bottom) on the temperature gradient. Error bars represent *SEM*. TTX indices are shown at the right side in boxplots. Difference between wild‐type and *nj24* mutants. *p < .05, **p < .01, ***p < .001, N.S. (not significant) by one‐way ANOVA followed by Tukey HSD test

To identify the molecular mechanisms underlying thermotaxis, a temperature learning food‐associated behavior, we have previously performed a forward genetic screen for mutants defective in thermotaxis and isolated *nj24* mutants (Mohri et al., 2005). To further characterize the *nj24* mutants, we conducted detailed thermotaxis behavior analysis in the population assays, which allowed us to assess the temperature to which animals migrate on a linear thermal gradient (Figure 1a) (Ito, Inada, & Mori, 2006). After cultivation at 17, 20, and 23°C with food, most of wild‐type animals migrated toward the past cultivation temperatures (Figure 1b,c). In contrast, *nj24* mutants cultivated at 20 and 23°C with food migrated to a temperature slightly higher than that of wild‐type animals. Thermophilic mutants reported to date tend to accumulate to the highest temperature on the thermal gradient and do not accumulate to the temperature slightly higher than cultivation temperature (Kuhara, Inada, Katsura, & Mori, 2002; Okochi, Kimura, Ohta, & Mori, 2005). Thus, the thermophilic behavior of *nj24* mutants is quite unique compared to other typical thermophilic mutants.

It is known that wild‐type animals disperse on the temperature gradient in association with past starved conditions (Nishio et al., 2012). We also determined whether the *nj24* mutation affects the thermotaxis behavior induced by starvation. Both the wild‐type and *nj24* mutants cultivated in food‐deprived condition lost the attraction to the cultivation temperature and dispersed on the thermal gradient (Figure 1b,c). Interestingly, in all cultivation temperature conditions tested, compared to wild‐type animals, the *nj24* mutant animals distributed to higher temperatures. These results indicate that *nj24* mutants tend to choose temperatures higher than wild‐type animals, regardless of attractive or aversive condition.

### 2.2 *nj24* mutants harbor a mutation in the *inx‐4* gene

**Figure 2:**
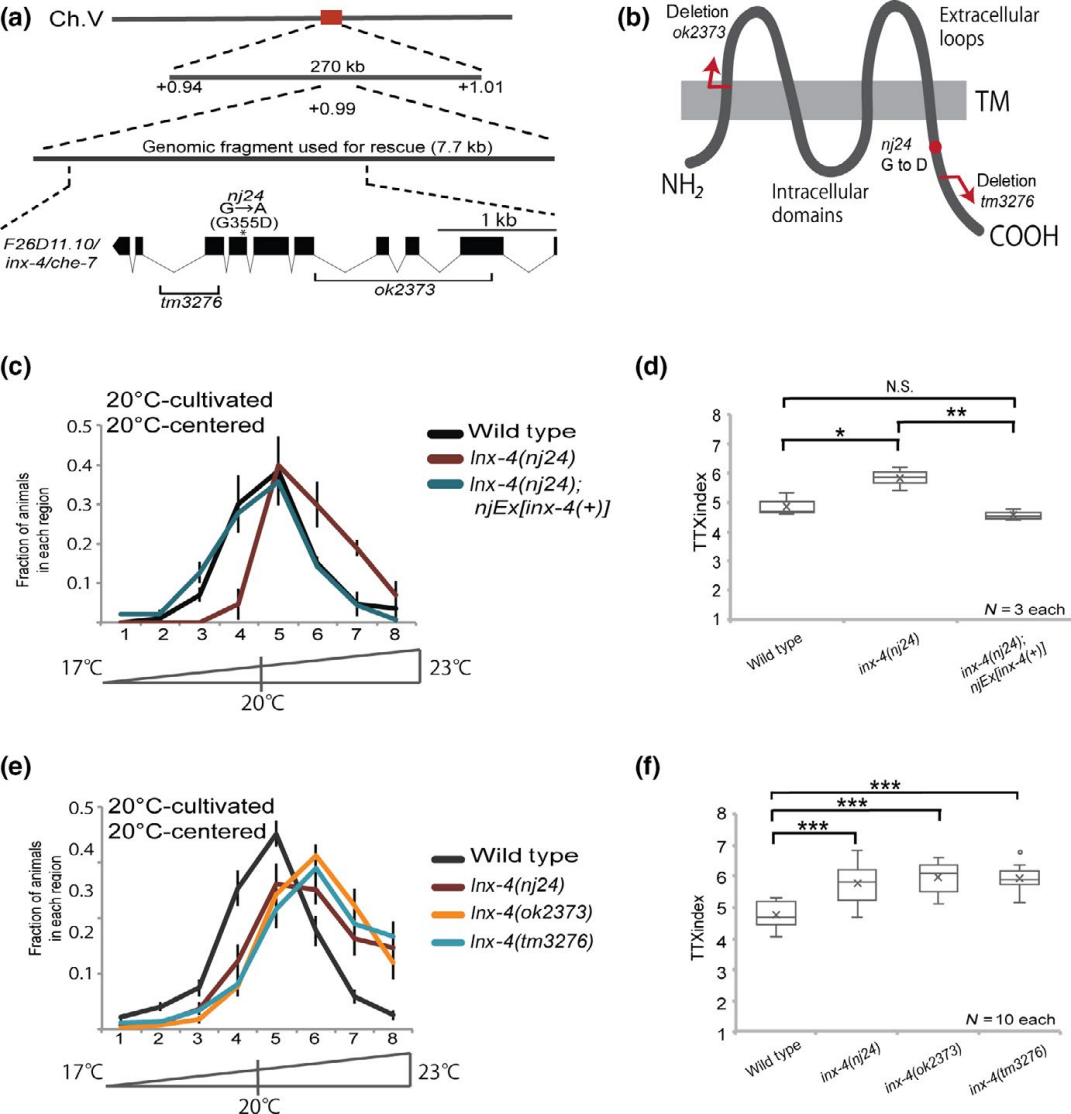
*inx‐4* is required for thermotaxis behavior. (a) The position and the predicted gene structure of *inx‐4* in LG V. The asterisk indicates the *nj24* mutation. The genomic regions deleted in *inx‐4(ok2373)* and *inx‐4(tm3276)* mutants are also indicated. (b) Predicted secondary structure of INX‐4 predicted by CCTOP. (c, d) Rescue experiment of *inx‐4(nj24)*. Boxplots for TTX indices are shown on the right. ***p < .001 by one‐way ANOVA followed by Tukey HSD test. (e, f) Thermotaxis of *inx‐4* mutants cultivated at 20°C. Boxplots for TTX indices are shown on the right. *p < .05, ***p < .001, N.S. (not significant) by one‐way ANOVA followed by Tukey HSD test

We then attempted to identify the mutation responsible for the *nj24* phenotype. SNP mapping indicated that the *nj24* mutation is in the + 0.94 to + 1.01 region of chromosome V. Subsequent sequence analysis revealed that the *nj24* mutant harbors a mis‐sense mutation (Gly355 to Asp) in the putative seventh exon of the *inx‐4* gene (Figure 2a and Figure S1). To confirm whether the thermal defects of *nj24* mutant were caused by the *inx‐4* mutation, we executed a rescue experiment by introducing a PCR product of *inx‐4* genomic DNA into the *nj24* mutant. The *nj24* mutant animals carrying the *inx‐4* genomic DNA exhibited behavior similar to the wild‐type animals (Figure 2c,d), suggesting that *nj24* is a mutation of the *inx‐4* gene.

The *inx‐4* gene encodes one of the innexin family proteins (Figure S1). Like the other innexin genes, *inx‐4* is predicted to have four transmembrane domains with intracellular N‐ and C‐termini (Confirmed by CCTOP: http://cctop.enzim.ttk.mta.hu). The *nj24* mutation corresponds to the intracellular C‐termini of INX‐4, which is predicted to locate in the cytoplasm (Figure 2b). To confirm that loss of the INX‐4 function also causes a phenotype similar to the *nj24* mutation, we tested the thermotaxis behavior of animals carrying deletions in the *inx‐4* gene. Both *inx‐4(ok2373)* and *inx‐4(tm3276)* mutants showed defects in thermotaxis behavior similar to *inx‐4(nj24)* mutants (Figure 2e,f), suggesting that *inx‐4* loss of function is the underlying cause for the impaired thermotaxis behavior of *nj24* mutants.

### 2.3 INX‐4 acts in AFD neuron to regulate thermotaxis behavior

**Figure 3:**
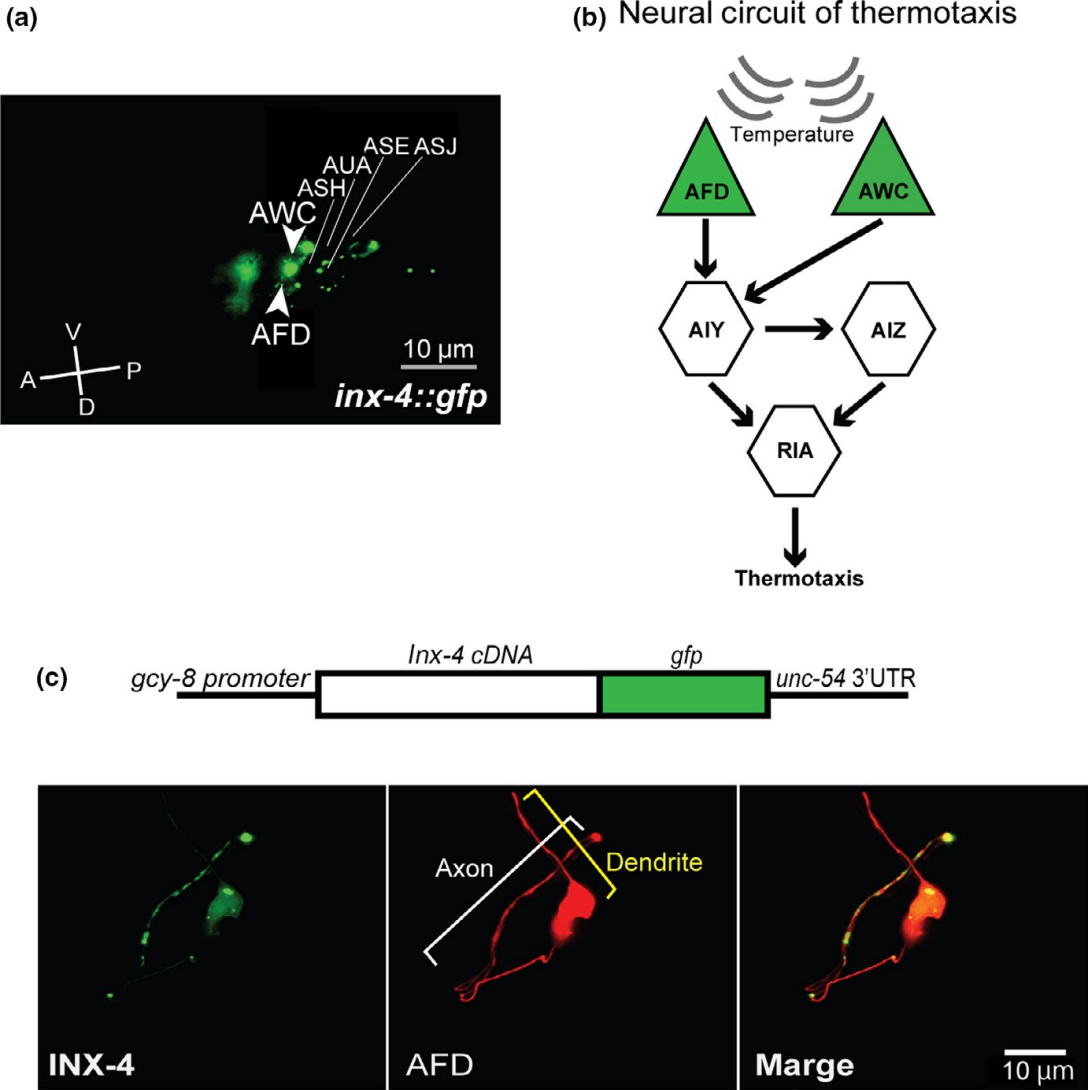
INX‐4 localized along the axon in AFD sensory neuron. (a) Expression of INX‐4 in the head region. Fluorescent images of animals carrying *inx‐4::gfp* transgene are shown. Several head neurons expressing INX‐4::GFP are indicated. Scale bar = 10 µm. (b) The proposed neural circuit for thermotaxis behavior (Kuhara et al., 2008; Mori & Ohshima, 1995). Neurons expressing *inx‐4::gfp* are colored in green. (c) Localization of INX‐4::GFP in AFD sensory neuron. Fluorescent images of animals carrying *gcy‐8p:: inx‐4::gfp* and *gcy‐8p:: tagrfp* are shown. The positions of an axon and a dendrite are indicated by white and yellow brackets, respectively. Scale bar = 10 µm

**Figure 4:**
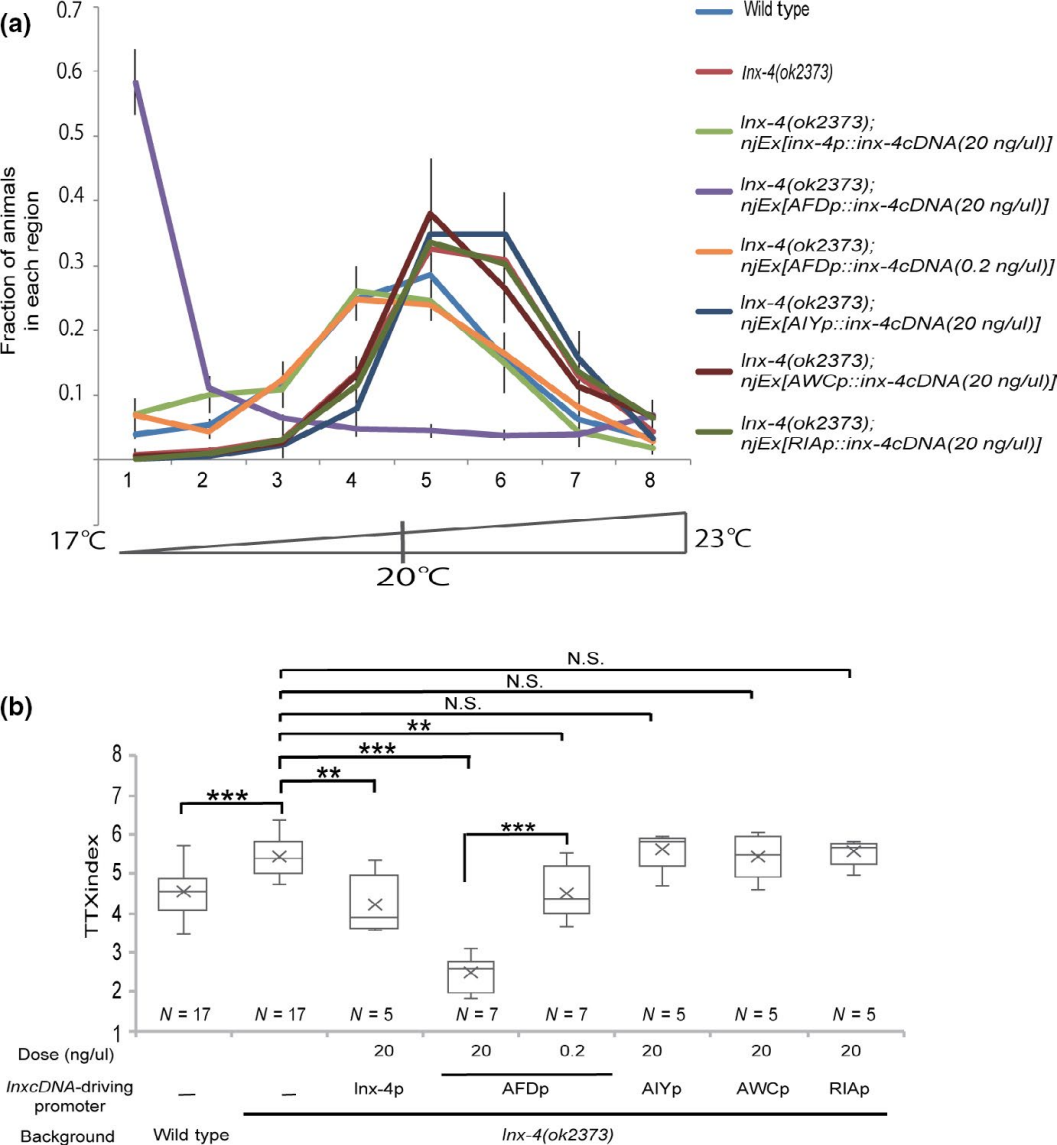
INX‐4 acts in AFD sensory neuron. (a) Cell‐specific rescue experiment of *inx‐4(ok2373)* mutants. Distributions of animals cultivated at 20°C on the temperature gradient. Error bars represent *SEM*. (b) TTX indices of the data shown in (a). **p < .01, ***p < .001, N.S. (not significant) by Kruskal–Wallis rank sum test followed by holm‐adjusted pairwise *t*‐test

To identify the cells expressing INX‐4, we constructed an *inx‐4* genomic DNA linked to the green fluorescent protein (GFP) gene and determined its expression pattern in animals. GFP fluorescence was observed predominantly in the nervous system, including AFD, AWC and ASE neurons (Figure 3a). Some of these neurons are known to act in the neural circuit of thermotaxis (Figure 3b). The transgene was able to rescue the thermotaxis defect of the *inx‐4* mutants (Figure S2a,b), indicating that GFP‐expressing cells include the cells involved in the regulation of thermotaxis behavior by INX‐4. To identify cell that requires INX‐4 to regulate thermotaxis behavior, we performed cell‐specific rescue experiment in *inx‐4(ok2373)* mutants. First, we expressed *inx‐4* cDNA by the *inx‐4* promoter and confirmed that it clearly rescued the thermotaxis defect of *inx‐4(ok2373)* mutants (Figure 4a,b). Next, we expressed INX‐4 using the *gcy‐8*, *AIY* (modified *ttx‐3*), *ceh‐36* or *glr‐3* promoter, which can express a transgene exclusively in AFD, AIY, AWC or RIA, respectively. We found that the expression of INX‐4 in AFD by the *gcy‐8p::inx‐4 cDNA* transgene (0.2 ng/μl) could rescue the defect of *inx‐4(ok2373)* mutants (Figure 4a,b). In contrast, expression of INX‐4 by the other promoters failed to rescue the thermotaxis defect of *inx‐4(ok2373)* mutants. These results indicate that INX‐4 acts in the AFD sensory neurons to regulate thermotaxis behavior. Interestingly, transgenic animals expressing INX‐4 in AFD at higher doses (20 ng/µl) showed a strong cryophilic phenotype (Figure 4a,b). This result suggests that over‐expression of INX‐4 in AFD causes the opposite phenotype to *inx‐4* mutants in thermotaxis behavior.

We further determined whether the abnormality of starvation‐induced thermotaxis behavior in *inx‐4(ok2373)* mutants can be rescued by the expression of INX‐4 in AFD neuron. The *inx‐4(ok2373)* animals carrying *gcy‐8p::inx‐4 cDNA* (0.2 ng/µl) transgene showed the starvation‐induced behavior similar to that of wild‐type animals (Figure S2c,d). These data indicate that INX‐4 acts in AFD in both attractive and aversive responses.

### 2.4 INX‐4 localizes exclusively along the axon of AFD neuron

We next attempted to examine the subcellular localization of INX‐4 protein in AFD neuron. We constructed *gcy‐8p::inx‐4 cDNA::gfp*, which produces the INX‐4::GFP fusion protein in AFD (Figure 3c) and introduced it at 2 ng/µl concentration in *inx‐4* mutants. In the transgenic strain, punctate localization of INX‐4::GFP was observed exclusively along the axon of AFD neuron (Figure 3c). To confirm that the transgene is functional, we executed rescue experiments using this DNA. Because the transgene introduced at 2 ng/µl concentration in animals caused the cryophilic phenotype (Figure S3), we introduced the same DNA at 0.2 ng/µl into the *inx‐4* mutant. The thermotaxis defect of *inx‐4* mutants was rescued by introducing the transgene (Figure S3), suggesting that the INX‐4::GFP fusion protein is functional. We could not use the rescued animal to determine the INX‐4::GFP localization because the GFP fluorescence was too weak (Data not shown).

### 2.5 Conserved Cys residues are required for the INX‐4 function

**Figure 5:**
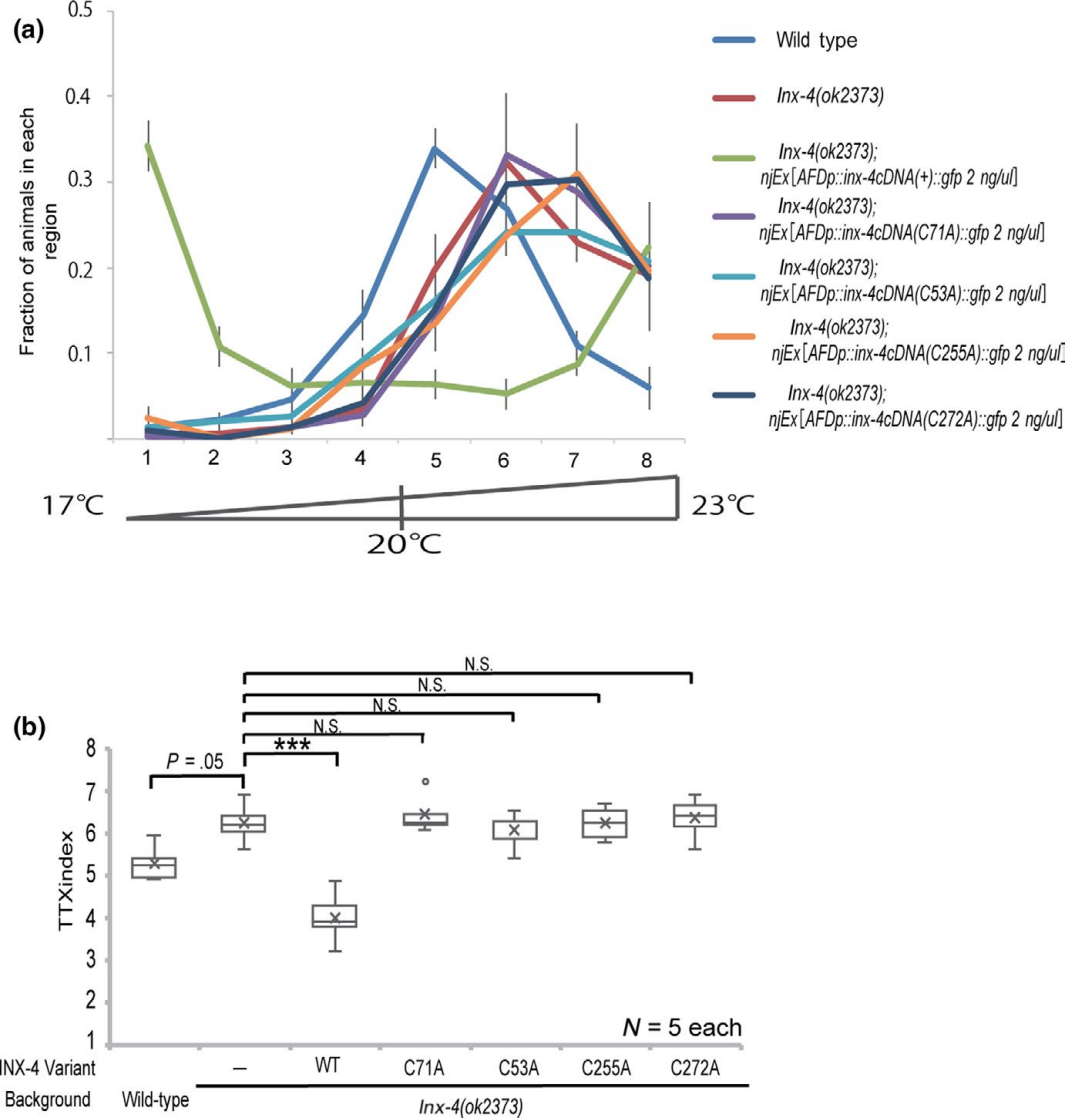
Conserved Cys residues are required for INX‐4 function. (a) Thermotaxis behavior of *inx‐4(ok2373)* expressing Cys‐less INX‐4::GFP in AFD. Error bars represent *SEM*. (b) Boxplots for TTX indices. ***p < .001, N.S. (not significant) by Kruskal–Wallis rank sum test followed by holm‐adjusted pairwise *t*‐test

Previous studies have revealed that cysteine residues at extracellular loops are well conserved among gap junction component proteins, including connexins, pannexins and innexins (Barbe et al., 2006). These Cys residues are also well conserved in *C. elegans* INX‐4 protein (Figure S1). In the case of mammalian connexin, lacking these Cys residues is known to disrupt the ability to form gap junction channels (Dahl, Werner, Levine, & Rabadan‐Diehl, 1992), but maintain ability to form functional hemichannel (Bao, Locovei, & Dahl, 2004; Elias, Wang, & Kriegstein, 2007). To test whether these conserved Cys in INX‐4 are important for its function, we constructed mutant forms of INX‐4 proteins, each of which carries a mutation that one of the four cysteines (Cys) is substituted to alanine (Ala). When each of these mutant protein was expressed in AFD of the *inx‐4* mutants at 2 ng/µl, all of them failed to alter the behavioral defect of *inx‐4* mutants, whereas expression of wild‐type INX‐4 form displayed a cryophilic behavior, presumably due to the over‐expression of the wild‐type INX‐4 (Figure 5a,b). These results suggest that the conserved Cys residues are essential for the regulation of thermotaxis behavior by INX‐4.

### 2.6 Calcium response of AFD was normal in *inx‐4* mutants

**Figure 6:**
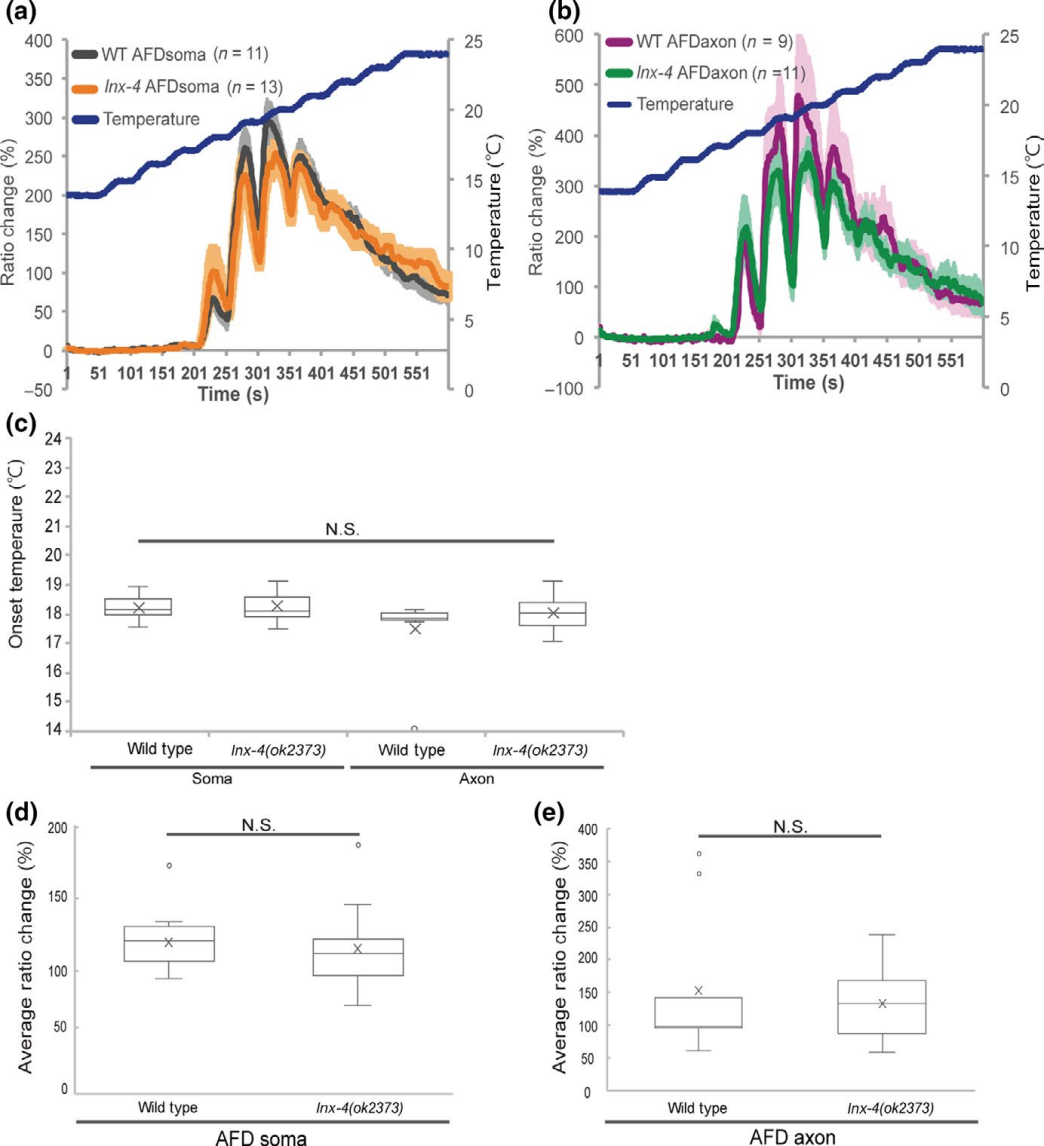
Calcium response of AFD in *inx‐4* mutants was normal. (a, b) Ratio changes of AFD calcium dynamics in wild‐type animals and *inx‐4(ok2373)* mutants. Colored lines represent mean values in each strain. Error bars represent *SEM*. (c) Boxplots of onset temperature (°C) for data shown in (a, b). Comparison between groups was performed by Kruskal–Wallis rank sum test. (d, e) Average ratio changes (%) between wild‐type animals and *inx‐4(ok2373)* mutants in AFD soma (d) and axon (e). Comparison between groups was performed by two‐sample *t*‐test for (d) and Wilcoxon rank sum test for (e)

Several studies have shown that calcium concentration of AFD soma increases around cultivation temperature in response to temperature warming (Clark, Biron, Sengupta, & Samuel, 2006; Kimura, Miyawaki, Matsumoto, & Mori, 2004; Kobayashi et al., 2016). The temperature that AFD calcium concentration began to increase varies depending on cultivation temperature, indicating that AFD can memorize cultivation temperature (Kimura et al., 2004; Kobayashi et al., 2016). As *inx‐4* mutants appeared to migrate toward the temperature slightly higher than the cultivation temperature (Figure 2e), we hypothesized that the temperature at which calcium begin to increase (defined as the onset temperature) might be shifted toward higher temperature in *inx‐4* mutants. We therefore examined calcium dynamics of AFD in *inx‐4* mutants using genetically encoded calcium indicator, GCaMP3. Unexpectedly, onset temperatures of calcium response were comparable between wild‐type animals and *inx‐4* mutants in AFD soma (Figure 6a,c). Moreover, as INX‐4 is expressed in the axon of AFD to regulate thermotaxis behavior (Figure 3c), we also assessed whether *inx‐4* mutants have altered local calcium dynamics in the axonal region. However, we could not detect significant difference in the onset temperatures between wild‐type and *inx‐4* mutants (Figure 6b,c). The average ratio change was also indistinguishable between wild‐type animals and *inx‐4* mutants in both AFD soma and axon (Figure 6d,e). These results suggest that INX‐4 acts downstream of calcium influx of AFD to regulate thermotaxis behavior.

## 3 DISCUSSION

Innexins are wide expressed in nervous system of *C. elegans* (Altun et al., 2009; Bhattacharya et al., 2019) to form gap junctions (electrical synapses). Some studies to date reported their implication of neuronal innexins in behavior: *unc‐9* for aggregation of social behavior (Jang et al., 2017), *inx‐1* for behavioral components of avoidance behavior (Hori, Oda, Suehiro, Iino, & Mitani, 2018), and *unc‐9* and *unc‐7* for bias for forward movement (Kawano et al., 2011) and regulation of sleep (Huang et al., 2018). Yet their roles to behavioral production are largely unknown.

In this study, we found that a type of innexin, INX‐4, is required for fine‐tuning of thermotaxis behavior. The behavior of *inx‐4* mutants is quite unique; they tend to move toward temperatures slightly higher than wild‐type animals, regardless of the feeding conditions. A plausible explanation for this observation is that *inx‐4* mutants misremember the cultivation temperature as temperature slightly higher than the actual value. Under this hypothesis, *inx‐4* mutants are less likely to migrate to higher temperature in aversive condition. However, both attractive and aversive behaviors of *inx‐4* mutants are thermophilic to the cultivation temperature. In addition, there was no significant difference in AFD calcium response between wild‐type and *inx‐4* mutant animals. These data indicate that the thermotaxis defects may occur at the point of the behavioral output, neither memory nor learning. Thus, *inx‐4* may be required for fine‐tuning execution of thermotaxis behavior, that is, moving toward desired temperatures.

In our study, the phenotype of *inx‐4* mutants was rescued by the expression of INX‐4 in AFD, indicating that INX‐4 acts in AFD neurons to regulate thermotaxis behavior. Localization of INX‐4::GFP along AFD axons implies that INX‐4 may act in the axon. As innexins seem to form gap junction channels (Hall, 2017; Simonsen et al., 2014), it is possible that INX‐4 also forms gap junctions along axons to regulate thermotaxis behavior. Consistent with this idea, conserved Cys residues, which are known to be required for formation of connexin gap junctional channels in mammals, are essential for the INX‐4 function in thermotaxis. Previous study has revealed that a pair of AFD neurons forms a gap junction at the end of their axons (White, Southgate, Thomson, & Brenner, 1986), raising the possibility that INX‐4 is required for electrical coupling between two AFDs. As neuronal cell coupling with gap junctions is known to attenuate noise and improve signal‐to‐noise ratio in mammalian retina (Söhl et al., 2005), the synchronized activation of two AFDs via INX‐4‐dependent electrical coupling might affect fine‐tuning of thermotaxis. In this scenario, INX‐4 helps to activate both AFDs simultaneously to guide the animals to reach the desired temperature.

Another possibility is that INX‐4 is required for the gap junctional connection(s) with other neurons in the regulation of thermotaxis. Although the classical wiring diagram in 1986 reported that left and right AFDs create gap junction circuits with each other and AIB (White et al., 1986), a recently re‐annotated wiring data showed that AFD has several additional gap junction partners, such as AIY, AWC, ADF, ASH, ASE, AVE and URB (Cook et al., 2019). Thus, INX‐4 in AFD can be involved in the formation of gap junction channels with these neurons. In addition to AFD, INX‐4 is expressed in other head sensory neurons including AWC and ASE, raising the possibility that INX‐4 forms homomeric gap junction channels with these neurons. However, our data clearly indicate that expression of INX‐4 only in AFD neurons is sufficient for the rescue of abnormal thermotaxis behavior. For this reason, INX‐4 might form heterotypic gap junctions with other type of innexin(s) if it functions as gap junction channels in thermotaxis behavior. As recent report indicates that *inx‐4* mutants also show defects in chemotaxis behavior to Na^+^, Cl^−^, Li^+^ (Smith et al., 2013) or quinine (Krzyzanowski et al., 2016), INX‐4 expressed in other neurons may function for other sensory behaviors.

Intriguingly, our result also implied that an appropriate expression level of INX‐4 in AFDs may be crucial to modulate thermotaxis behavior. The *inx‐4* mutants showed a mild thermophilic behavior, which could be rescued by introduction of appropriate amount of *inx‐4* gene expression. In contrast, over‐expression of INX‐4 in AFDs made animals to migrate to colder temperatures. Such dose‐dependent effect of INX‐4 on thermotaxis may not fit with the idea that INX‐4 functions by forming homomeric and heterotypic gap junction channels; it is difficult to explain that the increase of homomeric gap junction channels between a pair of AFDs causes the cryophilic phenotype, whereas heterotypic gap junction channels with other neurons may not drastically increase by the expression of INX‐4 in AFDs alone. We could also hypothesize that INX‐4 acts without forming of gap junction channels, as studies in mammalian model systems began to describe the physiological role of connexin or pannexin proteins as hemichannels in brain (Cheung, Chever, & Rouach, 2014). It would also be tempting to consider the possibility that INX‐4 acts as a hemichannel in AFDs.

In this study, we could not detect any defects of AFD calcium dynamics in *inx‐4* mutants. The onset temperature and average ratio change of wild‐type AFD were comparable to those of *inx‐4* mutants (Figure 6a–e). Although it is possible that our current imaging technique was not sufficient to detect the defect of *inx‐4* mutants in AFD as the thermophilic defect of *inx‐4* mutants was relatively mild, it is likely that calcium signals in AFD remain intact in *inx‐4* mutants, suggesting that INX‐4 may act in the downstream of calcium signaling of AFD. We believe further experiments will shed light on the mechanistic insights of the innexin family proteins in the sensory neurons to regulate behavior.

## 4 EXPERIMENTAL PROCEDURES

### 4.1 Strains

Strains were cultivated on nematode growth medium plates seeded with *E. coli* OP50 bacteria, essentially according to the basic methods (Brenner, 1974). The strains used in this study were listed in Table S1.

### 4.2 Thermotaxis behavioral analysis

The population thermotaxis assay was performed as previously reported (Ito et al., 2006; Nishio et al., 2012). Briefly, growth synchronized adults cultivated at 17, 20 and 23°C with OP50 were washed twice with NG buffer, then transferred to the middle line of the assay plate with thermal gradient and were allowed to move for 60 min. After 60 min, the animals were killed by chloroform gas. For evaluation of behavior analysis, the assay plate was divided into eight sections, and the number of animals in each section was counted. TTX index was calculated by a formula shown below. Starvation conditioning was conducted prior to assays as previously described (Nishio et al., 2012)*.*

$$TTXindex=\sum_{x=1}^{8}x\cdot Fx\;\left(Fx:\;\text{Fraction}\;\text{of}\;\text{animals}\;\text{in}\;\text{the}\;x\;\text{region},\;x=1-8\right)$$

### 4.3 Molecular biology and transgenic strains

For genomic DNA rescue experiment, the *inx‐4* genomic sequence including 2.8 kb upstream and 0.5 kb downstream region of *inx‐4a* coding sequence was amplified from N2 genome by PCR using following primers and introduced into *nj24* mutants.

F1: 5′‐ GTGTATTTGGCAGGCTGGAT ‐3′
R1: 5′‐ AGGTGCGACTAAAACCGACA ‐3′

For *inx‐4::gfp* construct, briefly, we amplified the *inx‐4* genomic sequence by PCR using following primers and inserted into pPD95.75.

F2: 5′‐ CCGCATGCGTGTATTTGGCAGGCTGGAT ‐3′
R2: 5′‐ CCGGATCCACTGCTAAAGGTACATTTT ‐3′

For cell‐specific rescue experiment, *cell‐specific promoter::inx‐4 cDNA* was generated. The *inx‐4* cDNA was amplified from the cDNA library, which was previously described (Sakamoto et al., 2005), using following primers.

F3: 5′‐ ATGGTGCTCCAGAATATG ‐3′
R3: 5′‐ CTATACTGCTAAAGGTAC ‐3′

Each *cell‐specific promoter::inx‐4 cDNA* plasmids was generated using following promoters, each of which drives expression exclusively in a single class of neurons: *gcy‐8p* for AFD, *AIYp* for AIY (modified *ttx‐3p*), *glr‐3p* for RIA, and *odr‐3p3* for AWC. Cell‐specific expressions driven by each promoter in targeted cells were confirmed previously (Kobayashi et al., 2016; Nishio et al., 2012; Tanizawa et al., 2006).

INX‐4 lacking extracellular cysteine residues (Cys‐less: C53A, C71A, C255A, C272A) was constructed by standard site‐directed mutagenesis method from *gcy‐8p::inx‐4 cDNA::gfp* plasmid (pEMM16). Four cysteines targeted are shown in Figure S1. The substituted mutation in each clone was confirmed by sequence analysis.

Transgenic lines were created by microinjection into the gonad syncytium (Mello, Kramer, Stinchcomb, & Ambros, 1991) and selected based on co‐injection marker, such as *ges‐1p::NLS‐GFP*, *ges‐1p::tagRFP* (See Table S1 for details). For all heterologous expression experiments, at least two independent transgenic lines were analyzed for more than one day.

### 4.4 Expression analysis of INX‐4

For cell identification in Figure 3a, animals carrying *inx‐4::gfp* were immobilized with 50 mM sodium azide. The identification was conducted by observing the positions and the size of the nuclei under BX53 microscopy (OLYMPUS) at L1‐L2 stage.

Subcellular localization of INX‐4 in AFD performed in Figure 3c was performed using LSM880 confocal microscopy (Zeiss). Adult animals cultivated at 20°C were immobilized with mixture of polystyrene beads and 50 mM sodium azide.

### 4.5 Calcium imaging analysis

Calcium imaging was performed essentially according to previous reports (Kobayashi et al., 2016; Ohnishi, Kuhara, Nakamura, Okochi, & Mori, 2011). Imaging was performed in animals expressing the genetically encoded calcium indicator GCaMP3 and tagRFP in AFD. Briefly, we prepicked 20°C‐cultivated L4 larval stage animals to synchronize the growth of the animals, and adults were imaged on the next day for the AFD soma and axon.

Individual animals were placed on a 10% agarose pad on a coverslip and immobilized by 0.1μm polystyrene beads (Polysciences), then covered with another coverslip. The samples were placed onto a Peltier‐based temperature controller (Tokai Hit), and image was captured by Olympus BX61WI microscope with 63x air objective lens using MetaMorph software (Molecular Devices) and an Image EM EM‐CCD camera (C9100‐13, Hamamatsu Photonics) at a rate of 1 Hz. The sample was initially kept at 14°C for 5 min and then subjected to the increasing temperature stimuli ranging from 14 to 24°C (Shown in Figure 6a,b).

Data analysis was performed with MetaMorph (Molecular Devices) and custom written scripts in AppleScript. Onset temperature was defined as the temperature at which ratio change (Δ*R*/*R* (%)) was increased over 50%. Average ratio change (%) in Figure 6d,e was defined as mean ratio change value from 51 to 550 s.

### 4.6 Statistical analysis

Error bars in all line graphs indicate the standard errors of the mean (*SEM*). Boxplots were created using Microsoft Excel. The “box” represents the middle 50% range of the data distributed between 1st and 3rd quartiles and divided into two parts by a line that indicates the median quartile. The top and bottom of the upper and lower whisker represent minimum and maximum, respectively. Dots represent outliers that are lower or higher than 1st quartile – the 1.5 times the interquartile range (IQR: middle 50% range) and 3rd quartile + the 1.5 times the IQR, respectively.

R studio software was used for all statistical tests. The Shapiro–Wilk normality test was used to test departures from normality. The Bartlett test was used to test homogeneity of variances. For those that *p*‐values were >.05 in both tests were followed by one‐way ANOVA with Tukey HSD test to examine statistical difference between groups. In case *p*‐value was <.05 in either normality test or homogeneity of variance test, nonparametric Kruskal–Wallis rank sum test was used. When *p*‐value is <.05 in the test, multiple comparison tests were performed by holm‐adjusted pairwise *t*‐test. For comparison between two groups in Figure 6d,e, two‐sample *t*‐test and Wilcoxon rank sum test were used, respectively. *p* < .05 are considered to be significant in all statistical tests (*: *p* < .05, **: *p* < .01, ***: *p* < .001).
