# Supplementary material for: The Caenorhabditis elegans INX‐4/Innexin is required for the fine‐tuning of temperature orientation in thermotaxis behavior
Source: Genes Cells. 2020 Jan 31;25(3):154–64. doi: 10.1111/gtc.12745 (PMC7078936; doi:10.1111/gtc.12745)
Supplement: Supplementary file 2 [file GTC-25-154-s002.pdf]

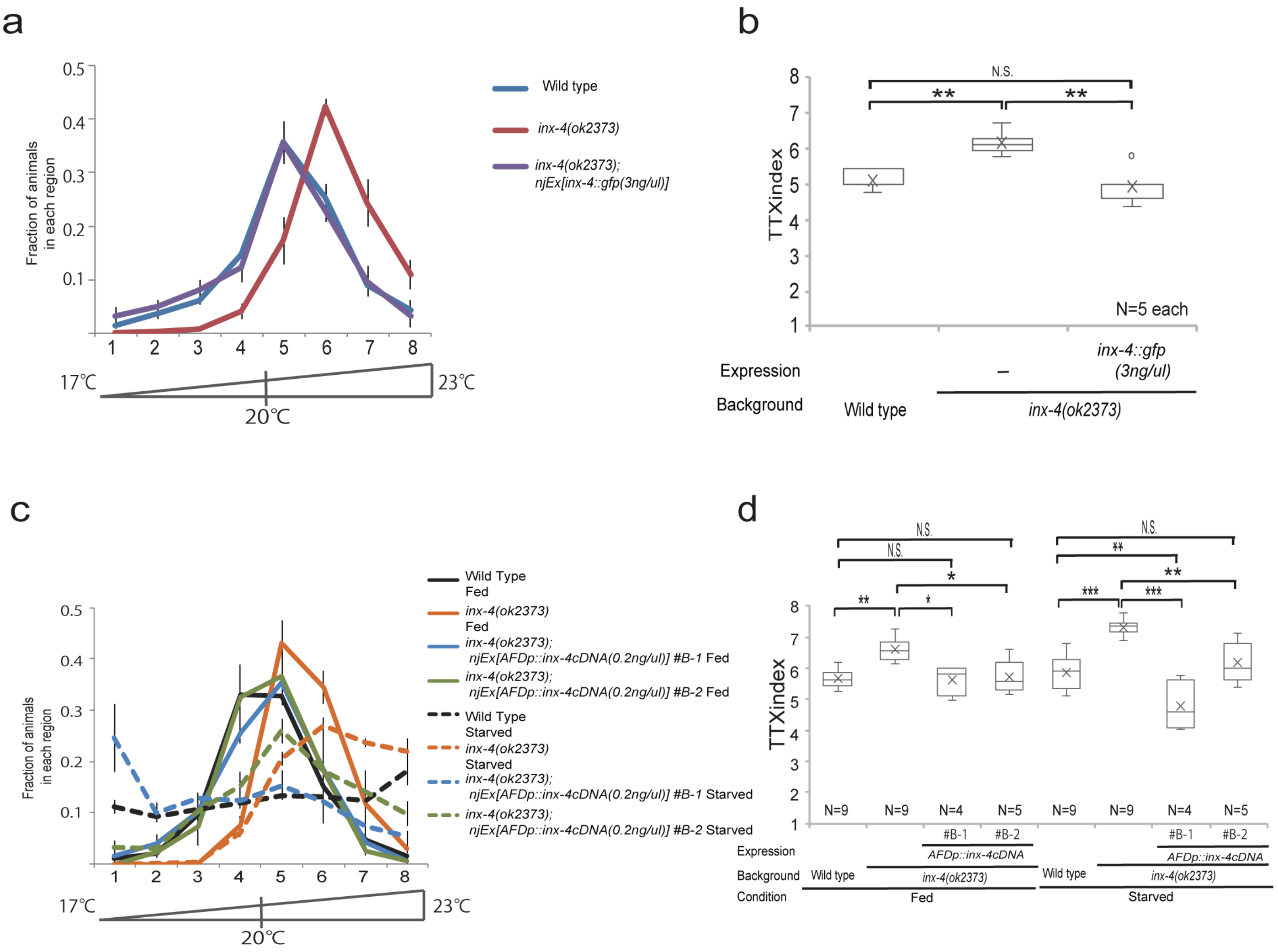

Figure S2. Rescue experiments of *inx-4(ok2373)* mutants. (A, C) Distributions of well-fed animals cultivated at 20°C along the temperature gradient. Error bars represent SEM.
(B, D) TTX indices of the data shown in (A, C), respectively. **P*<0.05, ***P*<0.01, ****P*<0.001 by one-way ANOVA followed by Tukey HSD test.
