# Supplementary material for: The Caenorhabditis elegans INX‐4/Innexin is required for the fine‐tuning of temperature orientation in thermotaxis behavior
Source: Genes Cells. 2020 Jan 31;25(3):154–64. doi: 10.1111/gtc.12745 (PMC7078936; doi:10.1111/gtc.12745)
Supplement: Supplementary file 4 [file GTC-25-154-s004.pdf]

Table S1. Strains used in this study.

Strain name	Genotype	Figures
N2	Wild-type Bristol strian	
IK2352	*inx-4(nj24)*	Fig2c-f
IK2432	*inx-4(nj24;); njEx[inx-4genomicDNA(2ng/ul), pKDK66 ges-1p::nls-gfp(75ng/ul)]#1*	Fig2c, d
IK2353	*inx-4(ok2373)*	Fig2e,f, 4a,b, 5a,b, FigS2a-d, FigS3 a,b
IK2354	*inx-4(tm3276)*	Fig2e,f
IK3567	*inx-4(ok2373); njEx1524[pST117.13 inx-4genomicDNA::gfp(20ng/ul), pNAS88 ges-1p::tagrfp(50ng/ul)]#2*	Fig3a
IK3568	*inx-4(ok2373); njEx1525[pEMM016 gcy-8p::inx-4cDNA::gfp(2ng/ul), pSAS318 gcy-8p::tagrfp(20ng/ul), pKDK66 ges-1p::nls-gfp(50ng/ul)]#1*	Fig.3c, FigS3. a-b
IK2453	*inx-4(ok2373); njEx962[pEMM010 inx-4p::inx-4cDNA(20ng/ul), pKDK66 ges-1p::nls-gfp(50ng/ul)]*	Fig.4a, b
IK2420	*inx-4(ok2373); njEx935[pEMM5 gcy-8p::inx-4cDNA(20ng/ul), pKDK66 ges-1p::nls-gfp(75ng/ul)]*	Fig.4a, b
IK3569	*inx-4(ok2373); njEx1526[pEMM5 gcy-8p::inx-4cDNA(0.2ng/ul), pKDK66 ges-1p::nls-gfp(50ng/ul)]#1*	Fig.4a, b
IK3570	*inx-4(ok2373); njEx1527[pEMM7 AIYp::inx-4cDNA(20ng/ul), pKDK66 ges-1p::nls-gfp(50ng/ul)]#1*	Fig.4a, b
IK3571	*inx-4(ok2373); njEx1528[pEMM8 ceh-36p3::inx-4cDNA(20ng/ul), pKDK66 ges-1p::nls-gfp(50ng/ul)]#1*	Fig.4a, b
IK3572	*inx-4(ok2373); njEx1529[pEMM14 glr-3p::inx-4cDNA(20ng/ul), pKDK66 ges-1p::nls-gfp(50ng/ul)]#1*	Fig.4a, b
IK3573	*inx-4(ok2373); njEx1530[pEMM016 gcy-8p::inx-4cDNA::gfp(2ng/ul), pNAS88 ges-1p::tagrfp(50ng/ul)]#B-1*	Fig.5a, b
IK3574	*inx-4(ok2373); njEx1531[pST124.3 gcy-8p::inx-4(C71A)::gfp(2ng/ul), pNAS88 ges-1p::tagrfp(50ng/ul)]#B-1*	Fig.5a, b
IK3575	*inx-4(ok2373); njEx1532[pST129.4 gcy-8p::inx-4(C53A)::gfp(2ng/ul), pNAS88 ges-1p::tagrfp(50ng/ul)]#1*	Fig.5a, b
IK3576	*inx-4(ok2373); njEx1533[pST130.1 gcy-8p::inx-4(C255A)::gfp(2ng/ul), pNAS88 ges-1p::tagrfp(50ng/ul)]#1*	Fig.5a, b
IK3577	*inx-4(ok2373); njEx1534[pST131.1 gcy-8p::inx-4(C272A)::gfp(2ng/ul), pNAS88 ges-1p::tagrfp(50ng/ul)]#1*	Fig.5a, b
IK961	*njIs24[gcy-8p::GCaMP3, gcy-8p::TagRFP]*	Fig.6 a-e
IK2464	*njIs24[gcy-8p::GCaMP3, gcy-8p::TagRFP]; inx-4(ok2373)*	Fig.6 a-e
IK3578	*inx-4(ok2373); njEx1535 [pST117.13 inx-4genomicDNA::gfp(3ng/ul), pNAS88 ges-1p::tagrfp(50ng/ul)]#10*	FigS2a-b
IK3579	*inx-4(ok2373); njEx1536[pEMM5 gcy-8p::inx-4cDNA(0.2ng/ul), pKDK66 ges-1p::nls-gfp(50ng/ul)] #B-1*	FigS2c-d
IK3580	*inx-4(ok2373); njEx1537 [pEMM5 gcy-8p::inx-4cDNA(0.2ng/ul), pKDK66 ges-1p::nls-gfp(50ng/ul)] #B-2*	FigS2c-d
IK3581	*inx-4(ok2373); njEx1538[pEMM016 gcy-8p::inx-4cDNA::gfp(0.2ng/ul), pSAS318 gcy-8p::tagrfp(20ng/ul), pKDK66 ges-1p::nls-gfp(50ng/ul)]#1*	FigS3a-b
