# Supplementary material for: The Caenorhabditis elegans INX‐4/Innexin is required for the fine‐tuning of temperature orientation in thermotaxis behavior
Source: Genes Cells. 2020 Jan 31;25(3):154–64. doi: 10.1111/gtc.12745 (PMC7078936; doi:10.1111/gtc.12745)
Supplement: Supplementary file 1 [file GTC-25-154-s001.pdf]

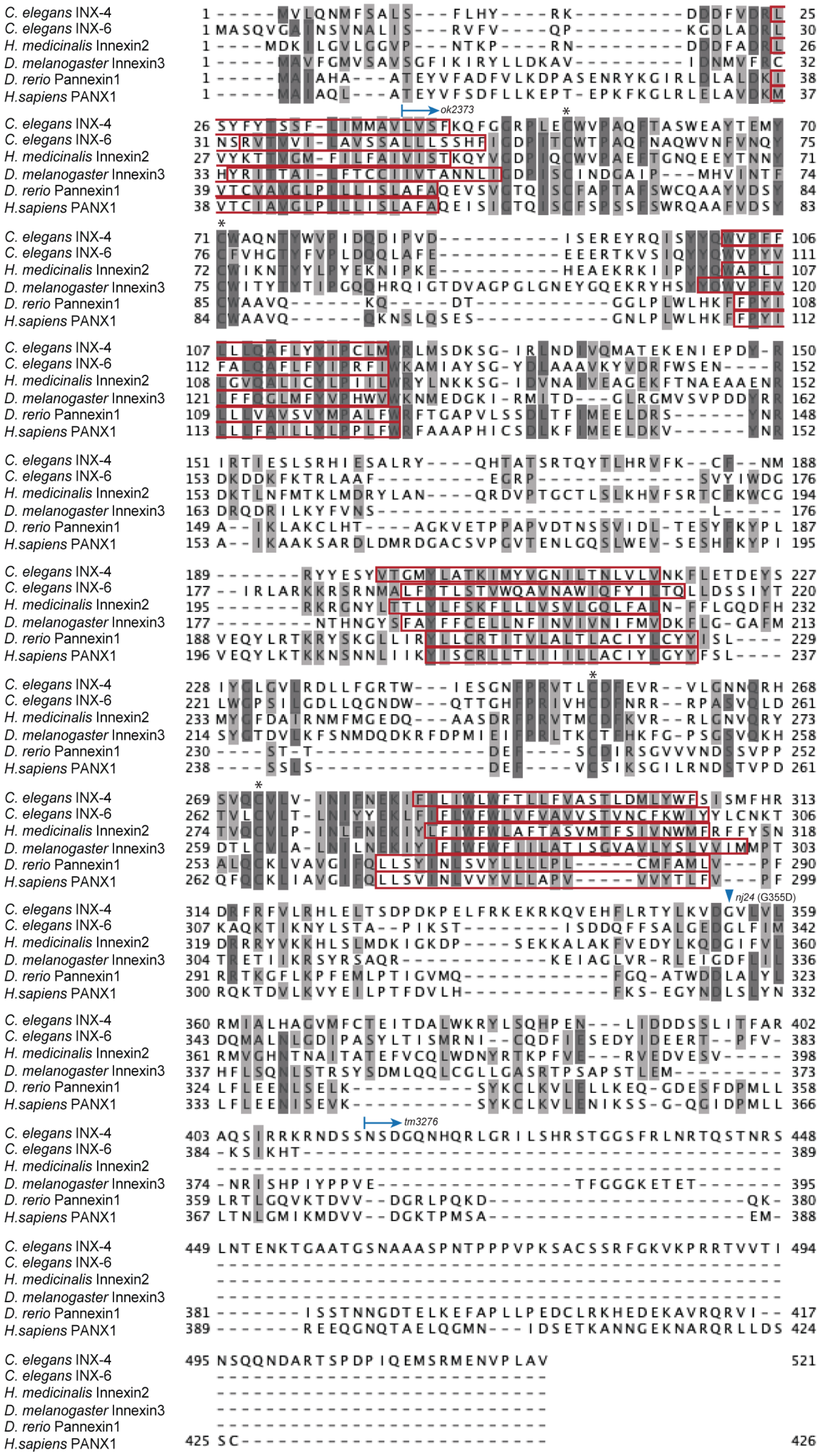

Figure S1. Sequence alignments of INX-4 and other innexins/pannexins. Identical and similar residues are highlighted with black and gray shading, respectively. The predicted transmembrane domains are indicated by red boxes. Conserved Cys residues targeted for mutagenesis in this study are indicated by asterisks. The *nj24* mutation and deletion sites of mutants are also shown.
